# A study of the effectiveness of the CBL combined with SP in the standardized training of general medicine residents

**DOI:** 10.3389/fmed.2026.1804359

**Published:** 2026-04-30

**Authors:** Meixia Xiao, Ziwei Lei, Yujian Gao, Yashu Wang, Shengming Shi

**Affiliations:** Department of General Practice, The First People's Hospital of Huzhou, First Affiliated Hospital of Huzhou University, Huzhou, China

**Keywords:** CBL, CBL-SP, clinical consultation skills, general practice residency training, SP

## Abstract

**Background:**

The standardized training of general practitioners is a crucial component of post-graduation medical education and a strategic initiative in China’s healthcare workforce planning. This study aimed to evaluate the effectiveness of an innovative teaching model— Case-Based Learning (CBL) combined with Standardized Patient (SP)—in the standardized training of general practice residents, with the goal of informing the development of general practitioner training and supporting national strategic health workforce policies.

**Methods:**

A total of 48 resident trainees participating in general practice standardized training in the First People’s Hospital of Huzhou City were randomly assigned to the CBL combined SP (CBL-SP) group under the CBL-SP model or the LBL group with traditional teaching. Assessments included knowledge tests, clinical consultation skills, evaluations of teaching satisfaction, conducted both before and after the general rotation were used to evaluate the teaching effects of the two groups.

**Results:**

Both groups demonstrated significant improvements in clinical competence scores. Comparing the general practice clinical competence of both groups before and after instruction, we found that the CBL-SP group achieved significantly greater improvements than the LBL group across stimulation of learning interest, subjective initiative in learning, knowledge extension ability, improvement in clinical reasoning skills, ability to guide healthy lifestyles, teamwork awareness, willingness to continue this teaching approach, and teaching satisfaction (*p* < 0.05).

**Conclusion:**

The CBL combined with SP demonstrates significant effectiveness in the standardized training of general practice residents. This innovative approach not only enhances learning outcomes and trainee satisfaction but also holds promise for supporting the strategic scaling of high-quality GP training across China, thereby contributing to the nation’s primary care workforce development goals.

## Introduction

As the “gatekeepers” of primary care, general practitioners must possess strong professional ethics and master the fundamental theories, knowledge, and skills of general medicine to independently conduct primary healthcare work. Standardized residency training in general practice is the key pathway for cultivating such versatile professionals, with its core objective being to enable physicians to systematically and competently address common community health issues (1). However, the current traditional teaching model, which emphasizes theoretical lectures and department rotations, makes it difficult for trainees to effectively integrate knowledge and apply clinical reasoning in complex clinical scenarios. It also lacks opportunities for repeated practice of core skills such as history-taking, physical examination, and communication in a safe, controlled environment (2). Faced with this situation, exploring and evaluating effective innovative teaching models has become an urgent task for deepening general practice medical education.

Case-Based Learning (CBL) is an innovative teaching model evolved from Problem-Based Learning (PBL). It represents a contextualized approach to learning and instruction that promotes active and reflective learning among trainees, fostering critical thinking and problem-solving skills (3). This methodology has gained widespread adoption in medical education globally (4). However, in traditional CBL models, cases are predominantly presented through written descriptions, lacking dynamic interactions with “real patients.” The Standardized Patient (SP) teaching method effectively addresses this limitation. By employing trained healthy individuals to simulate patients with specific conditions, SPs provide learners with a safe, realistic, and repeatable clinical practice environment. Research indicates that SPs significantly enhance learners’ communication skills, patient-centered care, and clinical reasoning abilities (5).

The combination of CBL and SP has emerged as a promising pedagogical strategy that synergistically integrates case-based reasoning with simulated clinical encounters. Several studies have demonstrated the efficacy of this combined approach in enhancing clinical competence across various medical disciplines. For instance, a randomized controlled trial by Zhou et al. (6) demonstrated that CBL combined with SP on a web-based platform significantly enhanced medical students’ clinical performance compared to traditional methods. A systematic review and meta-analysis by Chen et al. (7) further confirmed that CBL-SP integration yields positive effects on learners’ knowledge acquisition and clinical skills. Moreover, longitudinal research by Kim et al. (8) indicated that the benefits of CBL-SP training persist over time, enhancing both clinical competency and confidence. In psychiatric nursing education, Zhang et al. (9) reported that a blended learning approach incorporating CBL and SP improved students’ clinical skills and empathy.

Despite above promising findings, the existing literature predominantly focuses on undergraduate medical education or specialty-specific contexts such as psychiatry and nursing, leaving a critical gap in the domain of postgraduate general practice training. Moreover, most previous studies lack a robust theoretical or conceptual framework to explain why the CBL-SP model might be particularly effective for GP education, which emphasizes holistic, continuous, and patient-centered care.

From a theoretical perspective, the CBL-SP model aligns well with constructivist learning theory, which posits that learners actively construct knowledge through meaningful interactions with authentic contexts. By engaging trainees in realistic case scenarios and simulated patient encounters, the model fosters experiential learning—a process where knowledge is created through the transformation of experience (21). The iterative cycle of case analysis, simulated practice, and structured feedback mirrors the experiential learning cycle, thereby reinforcing deep understanding and skill acquisition. This theoretical grounding supports the rationale for applying CBL-SP in GP training, where learners must integrate biomedical knowledge with psychosocial considerations and communication skills in managing complex, real-world patient presentations.

Nevertheless, the application potential and practical outcomes of the CBL-SP model remain under-evaluated in the field of family medicine training. To date, no study has systematically investigated its effectiveness in the context of standardized residency training for general practitioners in China. This gap is particularly concerning given the national strategic priority to strengthen primary care workforce development. Therefore, this study aims to explore the implementation outcomes of CBL combined with SP teaching methods in standardized training for family medicine residents.

It seeks to fill the gap in practical research in this field and provide evidence-based support for innovating family medicine education models and cultivating high-quality talent. By grounding our investigation in experiential learning principles, we also contribute to the theoretical understanding of how integrated pedagogical approaches can enhance clinical competence in postgraduate medical education.

## Materials and methods

### Participants

A total of 50 participants were family medicine residents undergoing standardized training at the Department of General Practice, First People’s Hospital of Huzhou, from January 2022 to December 2025. All participants were medical graduates from various medical colleges and universities across China, all holding a bachelor’s degree in clinical medicine and having passed the national medical licensing examination prior to enrollment in the residency program.

The inclusion criteria were as follows: (1) voluntary participation in this study and signing the informed consent form; (2) full-time undergraduate graduation with a bachelor’s degree in clinical medicine; (3) no obvious physical or psychological abnormalities; and (4) no prior exposure to the CBL-SP integrated teaching model. The exclusion criteria were (1) involuntary participation in this study; and (2) poorly adherent participants. All responses were anonymized throughout the study. All methods were performed in accordance with the Declaration of Helsinki. After excluding residents who met excluded criteria, 96% (48 of 50) participated in the trial. The study was approved by the Institutional Ethics Committee of the First People’s Hospital of Huzhou City. Written informed consent was obtained from all participants.

A pilot study was conducted between September and November 2024, prior to the main trial, involving 10 residents (5 per group) to estimate the expected effect size for the primary outcome (clinical consultation skills). The pilot yielded a large effect size (Cohen’s d = 1.2). Given the small pilot sample, this estimate may be inflated. Therefore, a more conservative effect size (Cohen‘s d = 0.8) was adopted for the main trial’s sample size calculation. Using G*Power (version 3.1.9.7) for a two-tailed independent samples t-test with *α* = 0.05, power (1 − *β*) = 0.80, and Cohen‘s d = 0.8, the required sample size was 21 participants per group. Accounting for a 20% dropout rate, we aimed to recruit 26 participants per group. After excluding residents who met exclusion criteria, 96% (48 of 50) participated in the trial, achieving the target sample size.

### Randomization and study design

**Figure 1:**
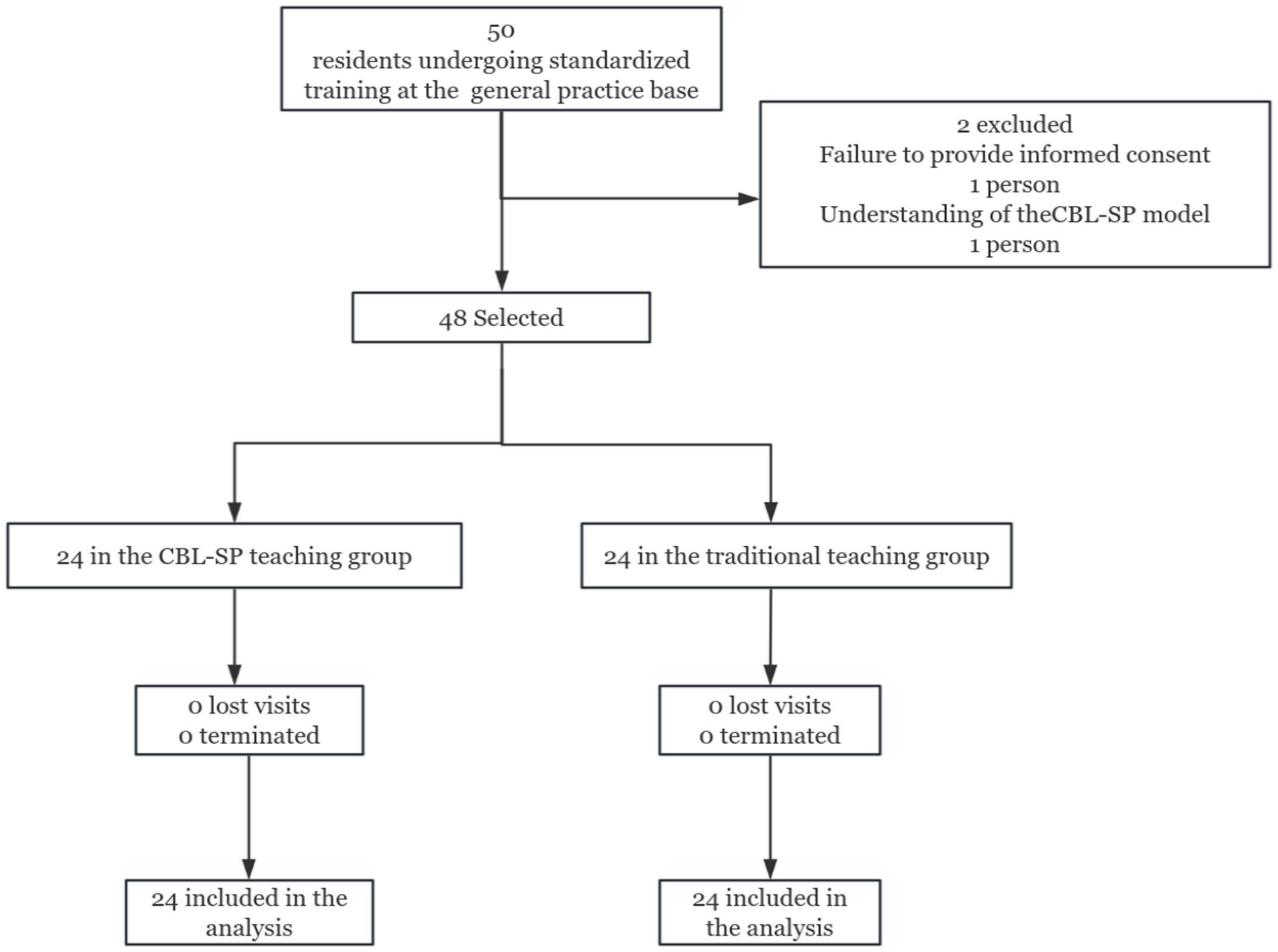
Flow chart of research subject enrollment. LBL, Traditional Teaching; CBL-SP, Case-Based Learning with Standardized Patients.

Participants were randomly assigned to either the CBL-SP teaching group or the traditional lecture-based learning (LBL) group (see Figure 1). An independent statistician not involved in the teaching or assessment processes generated a randomization list using SAS software (version 9.4, SAS Institute, Cary, NC, USA) with a 1:1 allocation ratio using simple randomization. The randomization sequence was concealed in sequentially numbered, opaque, sealed envelopes. The envelopes were opened by the study coordinator only after participants had completed baseline assessments and provided written informed consent. Neither the participants nor the instructors were aware of group assignments prior to the start of the intervention. Due to the nature of the educational interventions, blinding of participants and instructors during the implementation phase was not feasible; however, outcome assessors were blinded to group allocation.

### Research design

From January 1, 2025, a total of 48 residents were randomly assigned to either the LBL group (*n* = 24) or the CBL-SP group (*n* = 24) using a random number table method. The two groups underwent parallel, non-overlapping teaching interventions to minimize the risk of information cross-contamination. Participants were trained and evaluated separately, and they were explicitly instructed not to share teaching content between groups. Importantly, both groups of trainees undergo a standardized 12-week residency training in the Department of Family Medicine, including daily clinical training and two-hour weekly offline sessions; only the teaching approach differed between groups. Assessments included knowledge tests, clinical consultation skills, evaluations of teaching satisfaction, conducted both before and after the general rotation.

### Teaching implementation

#### LBL group

Participants are encouraged to preview relevant textbooks, consensus, and guidelines before the class, and the instructor conducted traditional classroom instruction based on the syllabus. The content and objectives of the LBL group are the same as those of the CBL-SP group. Both groups of trainees participated in normal ward work according to the “General Practice Residency Training Syllabus”, participants in the LBL group received conventional clinical teaching, which included daily theoretical instruction, clinical skills training, and a two-hour weekly offline session consisting of lectures, skill practices, teaching rounds, and medical record discussions.

#### CBL-SP group

**Figure 2:**
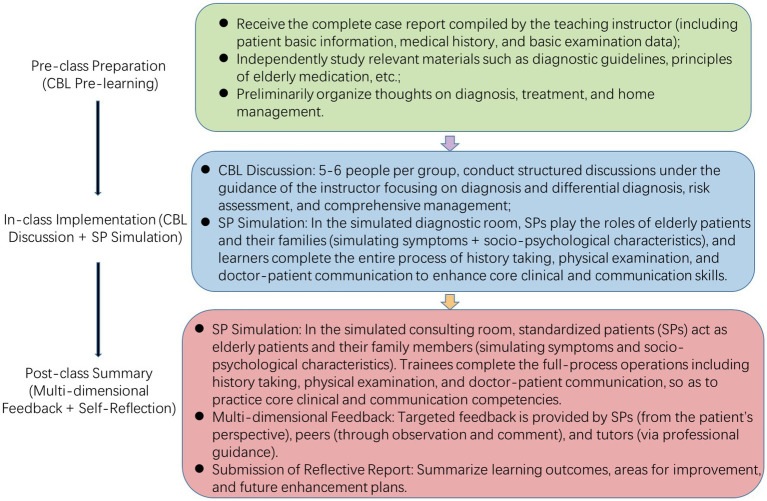
Taking the management of elderly patients with community-acquired pneumonia as an example.

Participants in the CBL-SP group received normal ward work with the same schedule and time allocation as the LBL group, including routine daily training and two-hour weekly offline sessions. In this group, offline courses employ the CBL-SP teaching approach, emphasis is placed on developing patient-centered general clinical reasoning, teamwork spirit, doctor-patient communication, and professional empathy. The teaching implementation for the CBL-SP group followed the structured three-phase model detailed in Figure 2.

#### CBL case study (pre-class + in-class)

##### Pre-class preparation

Trainees receive a comprehensive case report prepared by the supervising instructor, which includes basic information, preliminary medical history, and foundational examination data. Individuals should conduct independent study by reviewing the “Guidelines for the Diagnosis and Treatment of Community-Acquired Pneumonia,” principles for medication use in elderly patients, key points of home care, and other relevant materials. They should also preliminarily consider the diagnosis, differential diagnoses, treatment plan, and home management plan.

##### Classroom discussion

Students are divided into groups of 5–6 and engage in structured discussions guided by the instructor. Discussion Focus: Diagnosis and differential diagnosis, risk assessment, comprehensive management.

#### SP simulation practice (in-class)

##### Scenario setup

In a simulated examination room, trained Standardized Patients (SPs) portray the elderly patient and their family members. The SPs not only simulate symptoms but also vividly depict psychosocial characteristics such as loneliness from living alone, financial concerns, hearing loss, and cognitive impairment.

##### Student task

Complete the medical history interview, physical examination, doctor-patient communication, and outpatient medical record documentation to better demonstrate empathy, explanation of medical conditions, doctor-patient communication, and health education.

#### Structured feedback and summary (after class)

##### SP feedback

The SP provides feedback from the “patient’s” perspective: “Doctor, you explained it very clearly, but I’m still a bit worried about the medication being too expensive…” This directly trains the trainee’s ability to demonstrate humanistic care.

##### Peer feedback

Other group members provide comments based on their observations, highlighting strengths and areas for improvement to foster teamwork skills.

##### Mentor feedback

The instructor concluded with a summary feedback session, providing professional guidance based on direct observation regarding the logical flow of the consultation, the standardization of the physical examination, and the rationality of decision-making, thereby reintegrating practice with theory.

##### Self-reflection

Participants are required to submit a reflection report after the session, summarizing their key takeaways, areas for improvement, and future action plans.

### Effectiveness assessment

#### Academic performances

After and before the family medicine rotation, trainees from both groups took a theoretical examination in accordance with the Family Medicine Residency Training Syllabus. The theoretical test paper was compiled by the Family Medicine Teaching Secretary, scored out of 100 points, and reviewed by the Education and Training Department of Huzhou First People’s Hospital.

#### Clinical consultation skills

Before and after the teaching intervention, clinical consultation skills were objectively evaluated using the Leicester Assessment Package (LAP). LAP is a high-quality measurement tool for formative and summative assessment of general practitioners’ consultation skills, widely recognized and applied in European and American countries as well as Hong Kong. This assessment package comprises seven categories and 39 items: consultation and history taking, physical examination, patient management, problem solving, physician behavior and patient relationships, preventive care, and medical record keeping. All assessments were conducted by a single trained instructor who was blinded to group allocation. While LAP provides a structured framework for evaluation, the assessment of clinical consultation skills remains inherently subjective.

#### Teaching satisfaction

##### Teaching effectiveness satisfaction survey

An anonymous questionnaire assesses participants’ satisfaction with teaching outcomes. The survey employs a questionnaire independently designed for this study, covering eight dimensions: stimulation of learning interest, subjective initiative in learning, knowledge extension ability, improvement in clinical reasoning skills, ability to guide healthy lifestyles, doctor-patient communication, teamwork awareness, and willingness to continue this teaching approach. Each dimension is scored out of 10 points, with a total possible score of 80 points.

To establish the psychometric properties of this self-designed instrument, we assessed its content validity and internal consistency reliability. For content validity, the questionnaire was reviewed by three experts in general practice education, who evaluated the relevance of each item. The item-level content validity index (I-CVI) ranged from 0.83 to 1.00, and the scale-level content validity index (S-CVI/Ave) was 0.94, indicating good content validity. For internal consistency, the questionnaire demonstrated a Cronbach’s *α* coefficient of 0.87, exceeding the recommended threshold of 0.70 and indicating acceptable reliability.

#### Data evaluation and statistical analysis

Statistical analyses were performed using SPSS (version 22.0, IBM Corp., Armonk, NY, USA). Data normality was assessed using the Shapiro–Wilk test. Normally distributed continuous variables were expressed as mean ± SD, and compared using the independent samples t-test (between groups) or paired t-test (within groups). Non-normally distributed data were presented as median (IQR) and analyzed using the Mann–Whitney U test. Categorical variables were summarized as frequencies and percentages, and compared using the chi-square test. The magnitude of improvement (post-intervention score minus pre-intervention score) for each dimension of clinical consultation skills was calculated and compared between groups using the independent samples t-test. Statistical significance was set at *p* < 0.05 (two-tailed). A post-hoc power analysis was performed to assess the adequacy of the sample size for detecting differences in the primary outcome (clinical consultation skills). Based on the effect size calculated from the between-group comparison of total clinical consultation skill scores (Cohen’s d = 1.23), the achieved power was 0.94.

## Results

### Baseline comparison between the two groups

As shown in Table 1, the CBL-SP group comprised 24 trainees (13 females, 11 males) with a mean age of (23.16 ± 0.12) years. Their pre-instruction mean theoretical score was (76.53 ± 1.12), and mean clinical consultation competency score was (62.23 ± 1.23). The LBL group comprised 24 trainees (12 females, 12 males) with a mean age of (23.19 ± 0.17) years, pre-instructional mean theoretical scores of (76.26 ± 1.02), and mean clinical consultation ability scores of (62.17 ± 1.32). All participants in both groups held a bachelor’s degree in clinical medicine and had comparable educational and professional backgrounds prior to enrollment. Results indicated no significant differences in baseline data between the two groups (*p* > 0.05).

**Table 1:** Comparison of baseline data between the two groups of participants.

Characteristic	CBL-SP group (*n* = 24)	LBL group (*n* = 24)	*χ^2^*/*t* value	*p* value
Sex (male/female)	11/13	12/12	0.08	0.77
Average age (years)	23.16 ± 0.12	23.19 ± 0.17	0.71	0.48
Academic performances (score)	76.53 ± 1.12	76.26 ± 1.02	0.87	0.39
Total of clinical consultation skill (score)	62.23 ± 1.23	62.17 ± 1.32	0.16	0.87

All participants in both groups held a bachelor’s degree in clinical medicine and had comparable educational backgrounds prior to enrollment.

### Comparison of clinical consultation skills

Prior to instruction, the two groups of students showed no statistically significant differences in clinical competence scores across various dimensions. Following the teaching program, both groups demonstrated significant improvements in clinical competence scores across all dimensions. Comparing the general practice clinical competence of both groups before and after instruction, we found that the CBL-SP group achieved significantly greater improvements than the LBL group in most dimensions, including consultation and history taking, physical examination, patient management, problem solving, physician behavior and patient relationships, and medical record keeping (*p* < 0.05). However, no significant differences were observed in preventive care (*p* = 0.21). As shown in Table 2.

**Table 2:** Comparison of clinical consultation skills.

Skill dimension	CBL-SP group		*p*	LBL group		*p*	Difference value		*t*	*p*
	Before teaching	After teaching		Before teaching	After teaching		CBL-SP group	LBL group		
Consultation and history taking	7.41 ± 0.45	9.08 ± 0.62	0.001	7.31 ± 0.38	8.58 ± 0.63	0.001	2.23 ± 0.17	1.12 ± 0.12	26.74	<0.001
Physical examination	8.01 ± 0.65	8.49 ± 0.74	0.003	7.21 ± 0.68	7.65 ± 0.58	0.02	1.78 ± 1.12	1.02 ± 0.78	2.73	0.009
Patient management	7.43 ± 0.50	9.12 ± 0.56	0.001	7.56 ± 0.49	8.41 ± 0.84	0.001	2.01 ± 0.06	1.01 ± 0.26	18.77	<0.001
Problem solving	7.82 ± 0.24	9.34 ± 0.56	0.001	7.79 ± 0.37	8.67 ± 0.12	0.001	2.11 ± 0.12	1.12 ± 0.34	13.76	<0.001
Physician behavior and patient relationships	7.23 ± 0.45	8.89 ± 0.78	0.001	7.23 ± 0.98	8.12 ± 0.12	0.001	1.23 ± 0.12	0.99 ± 0.32	3.4	0.002
Preventive care	7.15 ± 0.68	8.98 ± 0.68	0.001	7.25 ± 0.78	8.23 ± 0.24	0.001	1.78 ± 0.15	1.34 ± 0.86	2.47	0.21
Medical record keeping	7.18 ± 0.65	9.21 ± 0.24	0.001	7.32 ± 0.76	8.56 ± 0.34	0.001	1.82 ± 0.23	1.12 ± 0.97	3.44	0.002

### Teaching satisfaction

Teaching satisfaction encompassed eight dimensions. The CBL-SP group achieved significantly higher scores than the LBL group in most dimensions, including stimulation of learning interest, subjective initiative in learning, knowledge extension ability, improvement in clinical reasoning skills, ability to guide healthy lifestyles, teamwork awareness, and willingness to continue this teaching approach (*p* < 0.05). However, no significant difference was observed in doctor-patient communication (*p* = 0.49). As shown in Table 3.

**Table 3:** Comparison of teaching satisfaction between groups.

Item	CBL-SP group (*n* = 24)	LBL group (*n* = 24)	*T* value	*p* value
Stimulation of learning interest	8.92 ± 0.45	7.23 ± 0.28	15.23	<0.001
Subjective initiative in learning	8.56 ± 0.75	7.66 ± 0.15	5.88	<0.001
Knowledge extension ability	8.56 ± 0.12	7.85 ± 0.98	3.52	0.002
Improvement in clinical reasoning skills	9.12 ± 0.16	7.56 ± 0.35	20.26	<0.001
Ability to guide healthy lifestyles	8.23 ± 0.15	7.89 ± 0.26	2.12	0.04
Doctor-patient communication	9.01 ± 0.12	8.63 ± 0.89	2.07	0.49
Teamwork awareness	8.35 ± 0.23	7.89 ± 0.88	2.48	0.02
Willingness to continue this teaching approach	9.23 ± 0.45	8.91 ± 0.12	3.37	0.002

## Discussion

This study addresses key challenges in traditional family medicine education by developing and evaluating an integrated CBL and SP clinical decision support teaching model for standardized training of family medicine residents. Our findings indicate that the CBL-SP integration significantly enhances general practice residents’ clinical reasoning abilities. Furthermore, this model markedly increases motivation for self-directed learning and yields higher teaching satisfaction compared to traditional CBL approaches, confirming its efficacy within standardized residency training programs. Notably, despite these overall positive findings, the improvements in preventive care (*p* = 0.21) and doctor-patient communication (*p* = 0.49) did not reach statistical significance, suggesting that the CBL-SP model may have limited advantage over traditional LBL in these specific dimensions.

With socioeconomic development and rising living standards, people’s demand for healthcare services continues to grow. Concurrently, factors such as industrialization, urbanization, and ecological changes increasingly impact health, while population aging and shifts in disease patterns present new challenges for healthcare delivery (10). In China, general practitioners serve as comprehensive healthcare professionals, undertaking the prevention, diagnosis, treatment, referral, rehabilitation, chronic disease management, and health management of common and prevalent illnesses within primary healthcare institutions (11).

However, the development of general practice residency training in China remains in its infancy, resulting in inadequate clinical practice training for general practitioners. Current training primarily relies on internships and observational learning, lacking opportunities for hands-on practice. It is not until the third year of residency training that general practitioners begin to independently manage patient care, typically within a narrow range of diseases. This lack of comprehensive clinical training hinders their ability to effectively handle diverse clinical scenarios (12, 13). Therefore, enhancing residents’ clinical practice capabilities is crucial for supporting their professional development. This need for improvement is a key reason for selecting this cohort as our study population.

Case-based teaching, utilizing authentic clinical cases as its vehicle, provides an effective pathway for standardized training of general practitioners (14). However, the success of this teaching model fundamentally depends on the quality and design of the cases themselves. In this study, all cases were meticulously selected and designed by experienced educators based on the “General Practice Residency Training Curriculum” and authentic clinical records from our hospital. Each case was specifically crafted to present common primary care issues, ensuring both educational relevance and clinical authenticity. This rigorous case development process is crucial for establishing a solid foundation for subsequent learning activities.

Case-based teaching, employs a closed-loop cycle model integrating case presentation with self-directed inquiry and instructor guidance, prompting trainees to actively synthesize core general practice knowledge. This includes the pathophysiology of common diseases, differential diagnosis, chronic disease management, and principles of preventive healthcare. Within GP training, this approach fosters collaborative discussions on common primary care health issues, enabling systematic learning from medical history analysis and differential diagnosis to developing long-term management plans. This process progressively helps trainees establish a complete clinical reasoning pathway from initial assessment to integrated management. Unlike traditional lecture-centered models—which often result in disconnects between theory and practice, inadequate clinical responsiveness, and flawed diagnostic logic—case-based teaching is learner-driven, emphasizing problem identification, analysis, and resolution (15).

While this approach may not comprehensively cover all syllabus-mandated knowledge points, its core value lies in cultivating general practitioners’ critical thinking, systematic history-taking skills, integrated judgment of multiple coexisting conditions, and evidence-based clinical decision-making capabilities Consequently, case-based teaching significantly enhances the quality of GP standardized training and trainees’ job competency. However, it is important to acknowledge that our experimental design introduced two concurrent variables—CBL and SP—within the intervention group. While CBL is no longer novel in clinical medical education, its combination with SP represents an innovative approach distinct from traditional methods.

Nevertheless, the relative contribution of each component remains unclear. It is possible that the observed benefits stem primarily from the SP component, from the enhanced CBL format, or from their synergistic interaction. To definitively isolate the effects of each teaching modality and further elucidate the advantages of this intervention, an ideal experimental design would include four parallel groups: CBL alone, SP alone, CBL-SP combined, and traditional LBL control. Such a design would allow for rigorous comparison of the independent and interactive effects of these educational strategies.

We found that the primary limitation of using CBL alone lies in its inability to simulate clinical scenarios, physician-patient communication, and the integration of theory and practice. Consequently, many scholars have begun combining CBL with other teaching methods, significantly enhancing educational outcomes (16–18). In contrast, SPs are trained individuals capable of consistently portraying specific medical conditions, symptoms, certain physical signs, and psychosocial characteristics within simulated clinical experiences. This approach provides learners with authentic, replicable interactions and quasi-realistic clinical scenarios within a controlled educational environment. It helps students enhance their patient communication skills and clinical competencies, effectively addressing the limitations of CBL. However, the primary constraint of SPs is their inability to simulate objective pathological signs.

Therefore, integrating CBL with SPs holds promise for further elevating the educational outcomes of clinical medical education. To date, research on integrated CBL + SP teaching methods remains scarce. This gap may stem from the substantial human resource investment required. In this study, students noted that the CBL + SP teaching model demands significant time and effort. The deep integration of CBL and SP essentially establishes a closed-loop educational system encompassing case cognition, scenario-based interaction, and structured feedback. It should also be noted that the assessment of clinical consultation skills in this study, while conducted using the validated LAP tool, relied on a single observer. Given the inherent subjectivity of such evaluations, the absence of multiple independent assessors may limit the reliability of the findings. Future studies should incorporate multiple trained observers and calculate inter-rater reliability to enhance the objectivity and robustness of skill assessments.

Teaching satisfaction serves as a crucial indicator for assessing the acceptance of educational reforms (19, 20). In this study, students in the CBL-SP group demonstrated significantly higher satisfaction with knowledge acquisition and achieved overall scores superior to those in the LBL group. These findings indicate strong student endorsement of the CBL-SP model. This approach effectively enhances students’ learning motivation and cultivates their self-directed learning abilities. Although it requires greater teacher investment in case design and classroom facilitation, the improvement in teaching outcomes is substantial. Implementing such educational innovations in standardized training for general practice residents represents a crucial strategy for enhancing their clinical competence and expanding the influence of the discipline of general practice.

An important consideration for the widespread adoption of the CBL-SP model is its resource implications compared to traditional LBL. In terms of time commitment, the CBL-SP model required approximately 4–6 h of preparation per case for instructors, including case development, SP training, and scenario design. This is substantially higher than the 1–2 h typically needed for preparing a traditional lecture. For trainees, while both groups participated in equivalent total instructional hours (two-hour weekly offline sessions), the CBL-SP group spent additional time on pre-class case preparation and post-session self-reflection, estimated at 2–3 h per week outside of formal sessions.

Regarding financial costs, the CBL-SP model incurred additional expenses for SP recruitment and training (approximately ¥100–200 per SP per session), as well as resources for simulated examination rooms and recording equipment. In contrast, the LBL group required only standard classroom facilities and instructor time. However, these initial investments should be weighed against potential long-term benefits. The enhanced clinical competencies and communication skills developed through CBL-SP may reduce supervision demands during clinical rotations and potentially improve patient outcomes in future practice. Furthermore, once cases and SP scripts are developed, they can be reused with minimal additional cost, making the model more cost-effective over time.

These resource considerations highlight an important trade-off: while the CBL-SP model demands greater upfront investment in time and financial resources, it yields superior educational outcomes. For training programs with limited resources, a hybrid approach—selectively applying CBL-SP to high-impact cases while maintaining LBL for routine content—may offer a pragmatic balance between educational quality and resource constraints.

Based on our experience and findings, we propose the following recommendations for effective implementation of the CBL-SP model in general practice residency training: (1) Structured case development. Develop cases from real clinical records aligned with the training syllabus, ensuring they reflect common primary care presentations and include clear learning objectives. (2) Standardized patient training. Train SPs thoroughly on clinical scenarios, symptoms, and relevant psychosocial characteristics. Regular refresher sessions maintain performance quality. (3) Multi-source feedback integration. Incorporate three levels of feedback: SP feedback (patient perspective), peer feedback (collaborative learning), and instructor feedback (clinical guidance). (4)Phased implementation for resource-limited programs. Start with high-impact cases while maintaining LBL for routine content. (5) Faculty development. Invest in training instructors, peer observation and mentoring support continuous improvement. (6) Curriculum integration.

## Limitations

Several limitations of this study should be acknowledged. First, although case-based learning (CBL) is no longer novel in clinical medical education, our study combined CBL with Standardized Patients (SP) to create an innovative approach distinct from traditional methods. However, the experimental group contained two concurrent variables—CBL and SP—making it impossible to determine which component contributed more significantly to the observed benefits. To further elucidate the advantages of this intervention, an ideal design would include four parallel groups (CBL alone, SP alone, CBL-SP combined, and traditional LBL control), enabling rigorous comparison of independent and interactive effects. Unfortunately, due to the finite number of residents enrolled in our program, this ideal four-group design was not feasible.

Additionally, the sample size calculation was based on a small pilot study (*n* = 10), which may have overestimated the true effect size. Although we adopted a more conservative effect size (*d* = 0.8) for the main trial, the possibility of residual inflation cannot be excluded. Furthermore, the teaching satisfaction questionnaire, while showing acceptable validity and reliability (CVI = 0.94, Cronbach’s *α* = 0.87), was self-designed and may benefit from further external validation.

Second, this was a single-center study with a limited sample size of 48 participants, which may restrict the generalizability of our findings to other training settings with different resident populations or institutional resources. Third, although participants were randomly assigned, participation was voluntary, which may introduce selection bias, as residents who agreed to enroll might differ from the broader trainee population in their motivation or openness to innovative teaching methods.

Last, while the Leicester Assessment Package (LAP) is a validated tool for assessing consultation skills, more authoritative and objective assessments—such as evaluations by blinded independent observers or objective structured clinical examinations (OSCEs)—would strengthen the validity of future research. Finally, the relatively short follow-up period limited our ability to assess long-term retention of clinical skills and their translation into independent practice. Larger-scale, multi-center studies with extended follow-up, multiple independent assessors, and blinded outcome evaluations are warranted to confirm and expand upon our findings.

## Conclusion

In conclusion, the CBL-SP model represents an effective and satisfactory approach to the standardized training of general practice residents. By fostering clinical thinking, self-directed learning, and practical skills, this model aligns with the goals of modern medical education and supports the strategic development of a competent general practitioner workforce in China. Widespread adoption and further evaluation of such innovative training methods are recommended to enhance the quality and scalability of general practitioner training in line with national health workforce planning.

## Data Availability

The original contributions presented in the study are included in the article/Supplementary material, further inquiries can be directed to the corresponding author.
