# Supplementary material for: A study of the effectiveness of the CBL combined with SP in the standardized training of general medicine residents
Source: Front Med (Lausanne). 2026 Apr 30;13:1804359. doi: 10.3389/fmed.2026.1804359 (PMC13171578; doi:10.3389/fmed.2026.1804359)
Supplement: Supplementary file 1 [file Table_1.DOCX]

**Questionnaire**

**Survey on Learning Effect and Satisfaction of General Practice Trainees in the First affiliated Hospital of Huzhou University**

Dear general practice trainees,

Thank you very much for your participation in our evaluation of teaching methods. Please evaluate the methods used in this course. There are eight questions.The questionnaire was assessed using a ten-point scale, where a score of 10 indicated “very satisfied” and a score of 1 denoted “very dissatisfied”, with intermediate values representing “relatively satisfied”, “generally satisfied”, and “not very satisfied”.

1. How do you evaluate this teaching method in improving your **subjective initiative in learning**?

Your answer is scores.

2.How do you evaluate this teaching method in improving your **knowledge expansion capabilities**?

Your answer is scores.

3.How do you evaluate this teaching method in improving your **enhancement of clinical reasoning skills**?

Your answer is scores.

4.How do you evaluate this teaching method in improving your **ability to guide healthy lifestyles**?

Your answer is scores.

5.How do you evaluate this teaching method in improving your **d**octor-patient communication?

Your answer is scores.

6.How do you evaluate this teaching method in improving your **teamwork awareness**?

Your answer is scores.

7.How do you evaluate this teaching method in improving your Stimulation of learning interest?

Your answer is scores.

8.How do you evaluate this teaching method in improving your  **willingness to continue adopting this teaching method**

?

Your answer is scores.
